# *N*,*N*′-Bis(2-aza­niumylbenz­yl)ethane-1,2-diaminium tetra­chloride

**DOI:** 10.1107/S1600536811045880

**Published:** 2011-11-09

**Authors:** Luis Ángel Garza Rodríguez, Sylvain Bernès, Perla Elizondo Martínez, Blanca Nájera Martínez, Nancy Pérez Rodríguez

**Affiliations:** a Laboratorio de Química Industrial, CELAES, Facultad de Ciencias Químicas, UANL, Pedro de Alba S/N, 66451 San Nicolás de los Garza, NL, Mexico; b DEP Facultad de Ciencias Químicas, UANL, Guerrero y Progreso S/N, Col. Treviño, 64570 Monterrey, NL, Mexico

## Abstract

The title compound, C_16_H_26_N_4_^4+^·4Cl^−^, is based on a fully protonated tetra­amine. In the cation, both benzene rings are connected by an all-*trans* chain, and the rings are almost parallel, with an angle between the mean planes of 8.34 (12)°. The benzene rings are arranged in such a way that the NH_3_^+^ substituents are oriented *cis* with respect to the central chain. This arrangement is a consequence of multiple N—H⋯Cl hydrogen bonds, involving all N—H groups in the cation and the four independent Cl^−^ anions. These contacts have strengths ranging from weak to strong (based on H⋯Cl separations), and generate a complex three-dimensional crystal structure with no preferential crystallographic orientation for the contacts.

## Related literature

For the structure of the free tetra­amine, see: Rodríguez de Barbarín *et al.* (2007 ▶). For related structures, see: Gakias *et al.* (2005 ▶); Garza Rodríguez *et al.* (2009 ▶, 2011 ▶). For the synthesis of the title hydro­chloride, see: Ansell *et al.* (1983 ▶); Gruenwedel (1968 ▶).
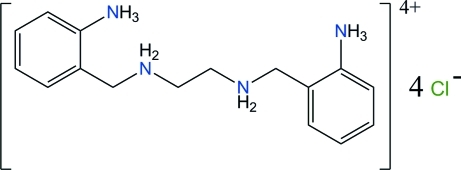


## Experimental

### 

#### Crystal data

C_16_H_26_N_4_^4+^·4Cl^−^
*M* *_r_* = 416.21
Triclinic, 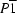

*a* = 8.6827 (13) Å
*b* = 11.4831 (17) Å
*c* = 11.7317 (17) Å
α = 117.773 (10)°
β = 101.826 (14)°
γ = 94.387 (16)°
*V* = 992.8 (3) Å^3^
*Z* = 2
Mo *K*α radiation
μ = 0.60 mm^−1^
*T* = 298 K
0.40 × 0.22 × 0.18 mm

#### Data collection

Siemens P4 diffractometer
Absorption correction: ψ scan (*XSCANS*; Siemens, 1996 ▶) *T*_min_ = 0.552, *T*_max_ = 0.607
6618 measured reflections
4022 independent reflections
3185 reflections with *I* > 2σ(*I*)
*R*_int_ = 0.029
2 standard reflections every 98 reflections  intensity decay: 1.5%

#### Refinement

*R*[*F* ^2^ > 2σ(*F* ^2^)] = 0.033
*wR*(*F* ^2^) = 0.090
*S* = 1.06
4022 reflections
219 parameters
H-atom parameters constrained
Δρ_max_ = 0.31 e Å^−3^
Δρ_min_ = −0.26 e Å^−3^

### 

Data collection: *XSCANS* (Siemens, 1996 ▶); cell refinement: *XSCANS*; data reduction: *XSCANS*; program(s) used to solve structure: *SHELXTL-Plus* (Sheldrick, 2008 ▶); program(s) used to refine structure: *SHELXTL-Plus*; molecular graphics: *SHELXTL-Plus* and *Mercury* (Macrae *et al.*, 2006 ▶); software used to prepare material for publication: *SHELXTL-Plus*.

## Supplementary Material

Crystal structure: contains datablock(s) I, global. DOI: 10.1107/S1600536811045880/fj2472sup1.cif

Structure factors: contains datablock(s) I. DOI: 10.1107/S1600536811045880/fj2472Isup2.hkl

Supplementary material file. DOI: 10.1107/S1600536811045880/fj2472Isup3.mol

Supplementary material file. DOI: 10.1107/S1600536811045880/fj2472Isup4.cml

Additional supplementary materials:  crystallographic information; 3D view; checkCIF report

## Figures and Tables

**Table 1:** Hydrogen-bond geometry (Å, °)

*D*—H⋯*A*	*D*—H	H⋯*A*	*D*⋯*A*	*D*—H⋯*A*
N1—H1*A*⋯Cl4	0.89	2.33	3.1067 (18)	146
N1—H1*B*⋯Cl1^i^	0.89	2.30	3.1798 (18)	170
N1—H1*C*⋯Cl2^ii^	0.89	2.26	3.1301 (18)	167
N9—H9*A*⋯Cl1	0.90	2.21	3.1046 (17)	172
N9—H9*B*⋯Cl2	0.90	2.22	3.0968 (17)	165
N12—H12*A*⋯Cl3	0.90	2.18	3.0333 (18)	159
N12—H12*B*⋯Cl2^iii^	0.90	2.35	3.1779 (17)	153
N20—H20*A*⋯Cl1^iv^	0.89	2.29	3.1764 (19)	173
N20—H20*B*⋯Cl3^iv^	0.89	2.56	3.2305 (18)	133
N20—H20*C*⋯Cl3	0.89	2.23	3.1139 (18)	173

Symmetry codes: (i) 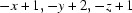; (ii) 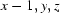; (iii) 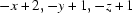; (iv) 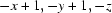.

**Fig. 1.**
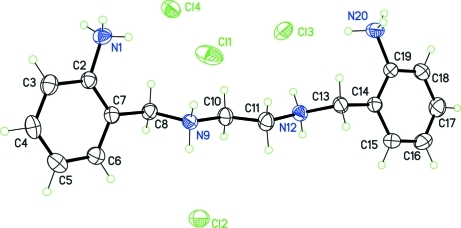
The structure of the title compound, with displacement ellipsoids at the 50% probability level.

**Fig. 2.**
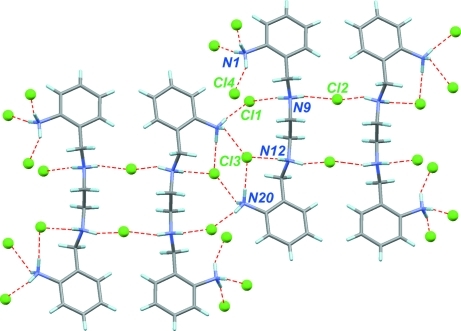
A part of the crystal structure of the title compound, showing the hydrogen bonds (dashed lines) between NH groups in cations and chloride ions (represented as green spheres). N and Cl atoms are labeled for the asymmetric unit.

## supplementary crystallographic information

### Comment

The tetraamine *N*,*N*'-bis(2-aminobenzyl)ethane-1,2-diamine, C_16_H_22_N_4_, is a tetradentate ligand for transition metals (Rodríguez de Barbarín *et al.*, 2007) and a precursor to the corresponding Schiff base *N*,*N*'-bis(2-aminobenzylidene)ethane-1,2-diamine (C_16_H_18_N_4_, Gakias *et al.*, 2005). Both are potentially useful as building blocks for the synthesis of macrocyclic ligands. However, we detected that these small amines and other related polyamines are frequently protonated if workup is carried out in acid media. If stabilizing anions are available in solution, a very stable salt is formed, which crystallizes readily, impeding the formation of the desired macrocycle. In view of this behavior, it is important to characterize as many salts as possible, in order to avoid anions which are prone to compete with the macrocycle synthesis. Some cationic species formed from the above quoted tetraamine C_16_H_22_N_4_ have been stabilized, for instance, with a mixture of nitrate and perchlorate (Garza Rodríguez *et al.*, 2009) and with tosylate (Garza Rodríguez *et al.*, 2011).

The title compound is the tetrahydrochloride salt of C_16_H_22_N_4_. In the cation, all amine groups are protonated, and the charges are balanced by four Cl^-^ anions (Fig. 1). In contrast with the free amine (Rodríguez de Barbarín *et al.*, 2007), the cation lies in general position. Another difference with the free amine is the conformation of the central chain linking the benzene rings. In the title compound, the chain is extended in the all-*trans* conformation, as reflected by torsion angles C7—C8—N9—C10 = -177.27 (15)°, C8—N9—C10—C11 = 172.08 (16)°, N9—C10—C11—N12 = -175.50 (15)°, C10—C11—N12—C13 = 162.72 (16)°, and C11—N12—C13—C14 = 169.22 (16)°. The benzene rings, although not related by symmetry, are almost parallel: the dihedral angle between their mean planes is 8.34 (12)°. A feature not observed in other related salts is the arrangement of ammonium NH_3_^+^ groups: one benzene group is rotated in order to place the NH_3_^+^ functionalities *cis* with respect to the central chain (see Fig. 1).

The *cis*-NH_3_^+^ conformation is very probably a consequence of the crystal structure, dominated by N—H···Cl hydrogen bonds. All ammonium H atoms and chloride Cl^-^ anions participate in the hydrogen bonds framework (Fig. 2), affording a complex three-dimensional crystal structure. The range for H···Cl separations is from 2.18 to 2.56 Å and N—H···Cl angles are in the range 133.2–173.3°, indicating that all contacts significantly participate in the stabilization of the crystal structure.

### Experimental

The title salt was obtained while attempting to dissolve a macrocycle in ethanol. *N*,*N*'-bis(2-aminobenzyl)ethane-1,2-diamine (50 mg, 0.185 mmol), 2,6-diacetylpyridine (30 mg, 0.185 mmol) and a concentrated solution of HCl (0.1 mmol) were mixed in 3.5 ml of ethanol. After 10 days of slow evaporation, crystals were formed and separated (7 mg of the title salt). Direct synthetic routes may also be found in the literature (Ansell *et al.*, 1983; Gruenwedel, 1968).

### Refinement

All C-bonded H atoms were placed in calculated positions, with C—H bond lengths fixed to 0.93 (aromatic CH), or 0.97 Å (methylene CH_2_) and *U*_iso_(H) = 1.2*U*_eq_(C). N-bonded H atoms were detected in a difference map, corroborating that all N atoms are protonated in the cation. They were placed in idealized positions, with N—H = 0.89 Å for NH_3_ and N—H = 0.90 Å for NH_2_ groups. The NH_3_ were considered as rigid groups but were allowed to rotate about their C—N bonds. Isotropic displacement parameters for these H atoms were calculated as *U*_iso_(H) = 1.2*U*_eq_(N) for NH_2_ and *U*_iso_(H) = 1.5*U*_eq_(N) for NH_3_.

### Crystal data

**Table:**

C_16_H_26_N_4_^4+^·4Cl^−^	*Z* = 2
*M**_r_* = 416.21	*F*(000) = 436
Triclinic, *P*1	*D*_x_ = 1.392 Mg m^−^^3^
Hall symbol: -P 1	Mo *K*α radiation, λ = 0.71073 Å
*a* = 8.6827 (13) Å	Cell parameters from 100 reflections
*b* = 11.4831 (17) Å	θ = 4.8–12.4°
*c* = 11.7317 (17) Å	µ = 0.60 mm^−^^1^
α = 117.773 (10)°	*T* = 298 K
β = 101.826 (14)°	Irregular, yellow
γ = 94.387 (16)°	0.40 × 0.22 × 0.18 mm
*V* = 992.8 (3) Å^3^	

### Data collection

**Table:**

Siemens P4 diffractometer	3185 reflections with *I* > 2σ(*I*)
Radiation source: fine-focus sealed tube	*R*_int_ = 0.029
graphite	θ_max_ = 26.4°, θ_min_ = 2.0°
ω scans	*h* = −10→5
Absorption correction: ψ scan (*XSCANS*; Siemens, 1996)	*k* = −13→13
*T*_min_ = 0.552, *T*_max_ = 0.607	*l* = −14→14
6618 measured reflections	2 standard reflections every 98 reflections
4022 independent reflections	intensity decay: 1.5%

### Refinement

**Table:**

Refinement on *F*^2^	Primary atom site location: structure-invariant direct methods
Least-squares matrix: full	Secondary atom site location: difference Fourier map
*R*[*F*^2^ > 2σ(*F*^2^)] = 0.033	Hydrogen site location: inferred from neighbouring sites
*wR*(*F*^2^) = 0.090	H-atom parameters constrained
*S* = 1.06	*w* = 1/[σ^2^(*F*_o_^2^) + (0.0329*P*)^2^ + 0.4207*P*] where *P* = (*F*_o_^2^ + 2*F*_c_^2^)/3
4022 reflections	(Δ/σ)_max_ = 0.001
219 parameters	Δρ_max_ = 0.31 e Å^−^^3^
0 restraints	Δρ_min_ = −0.26 e Å^−^^3^
0 constraints	

### Fractional atomic coordinates and isotropic or equivalent isotropic displacement parameters (Å^2^)

**Table:**

	*x*	*y*	*z*	*U*_iso_*/*U*_eq_	
Cl1	0.64430 (7)	0.88174 (6)	0.41424 (6)	0.04766 (16)	
Cl2	1.08774 (6)	0.74843 (6)	0.69409 (5)	0.03914 (14)	
Cl3	0.45844 (6)	0.37199 (6)	0.07026 (6)	0.04107 (14)	
Cl4	0.25755 (6)	0.58183 (5)	0.34958 (5)	0.03896 (14)	
N1	0.36517 (19)	0.85610 (16)	0.61340 (16)	0.0315 (4)	
H1A	0.3793	0.7855	0.5426	0.047*	
H1B	0.3539	0.9227	0.5954	0.047*	
H1C	0.2774	0.8333	0.6324	0.047*	
C2	0.5048 (2)	0.90023 (19)	0.72836 (18)	0.0273 (4)	
C3	0.5098 (2)	1.0198 (2)	0.8409 (2)	0.0331 (4)	
H3A	0.4282	1.0676	0.8404	0.040*	
C4	0.6361 (3)	1.0679 (2)	0.95391 (19)	0.0357 (5)	
H4A	0.6396	1.1480	1.0301	0.043*	
C5	0.7571 (3)	0.9971 (2)	0.9539 (2)	0.0389 (5)	
H5A	0.8440	1.0303	1.0293	0.047*	
C6	0.7488 (2)	0.8769 (2)	0.8416 (2)	0.0346 (4)	
H6A	0.8301	0.8290	0.8430	0.042*	
C7	0.6222 (2)	0.82504 (19)	0.72644 (18)	0.0267 (4)	
C8	0.6184 (2)	0.68997 (19)	0.61126 (19)	0.0288 (4)	
H8A	0.5107	0.6538	0.5511	0.035*	
H8B	0.6449	0.6291	0.6448	0.035*	
N9	0.73471 (18)	0.69858 (16)	0.53534 (15)	0.0264 (3)	
H9A	0.7130	0.7584	0.5081	0.032*	
H9B	0.8349	0.7287	0.5905	0.032*	
C10	0.7277 (2)	0.5675 (2)	0.41706 (19)	0.0324 (4)	
H10A	0.6176	0.5286	0.3625	0.039*	
H10B	0.7667	0.5060	0.4461	0.039*	
C11	0.8301 (2)	0.5883 (2)	0.33596 (19)	0.0328 (4)	
H11A	0.7965	0.6555	0.3135	0.039*	
H11B	0.9415	0.6209	0.3886	0.039*	
N12	0.81500 (19)	0.46002 (16)	0.21081 (15)	0.0279 (3)	
H12A	0.7116	0.4172	0.1753	0.034*	
H12B	0.8728	0.4068	0.2305	0.034*	
C13	0.8726 (2)	0.48425 (19)	0.11018 (18)	0.0284 (4)	
H13A	0.9760	0.5452	0.1541	0.034*	
H13B	0.7979	0.5273	0.0770	0.034*	
C14	0.8885 (2)	0.35751 (19)	−0.00620 (18)	0.0267 (4)	
C15	1.0288 (2)	0.3081 (2)	0.0064 (2)	0.0326 (4)	
H15A	1.1068	0.3510	0.0883	0.039*	
C16	1.0547 (3)	0.1976 (2)	−0.0991 (2)	0.0392 (5)	
H16A	1.1503	0.1675	−0.0886	0.047*	
C17	0.9393 (3)	0.1313 (2)	−0.2207 (2)	0.0409 (5)	
H17A	0.9566	0.0561	−0.2919	0.049*	
C18	0.7979 (3)	0.1767 (2)	−0.2363 (2)	0.0351 (5)	
H18A	0.7195	0.1322	−0.3179	0.042*	
C19	0.7740 (2)	0.28834 (19)	−0.13001 (18)	0.0273 (4)	
N20	0.62423 (19)	0.33434 (17)	−0.15287 (16)	0.0337 (4)	
H20A	0.5555	0.2703	−0.2285	0.051*	
H20B	0.6441	0.4080	−0.1592	0.051*	
H20C	0.5819	0.3528	−0.0848	0.051*	

### Atomic displacement parameters (Å^2^)

**Table:**

	*U*^11^	*U*^22^	*U*^33^	*U*^12^	*U*^13^	*U*^23^
Cl1	0.0568 (3)	0.0335 (3)	0.0427 (3)	0.0081 (2)	−0.0071 (2)	0.0193 (2)
Cl2	0.0314 (3)	0.0470 (3)	0.0387 (3)	0.0066 (2)	0.0051 (2)	0.0229 (2)
Cl3	0.0302 (3)	0.0403 (3)	0.0467 (3)	0.0091 (2)	0.0124 (2)	0.0157 (2)
Cl4	0.0450 (3)	0.0356 (3)	0.0309 (3)	0.0085 (2)	0.0039 (2)	0.0146 (2)
N1	0.0307 (8)	0.0285 (9)	0.0344 (9)	0.0088 (7)	0.0062 (7)	0.0155 (7)
C2	0.0294 (9)	0.0283 (10)	0.0267 (9)	0.0065 (8)	0.0096 (7)	0.0146 (8)
C3	0.0380 (11)	0.0281 (10)	0.0352 (11)	0.0099 (8)	0.0145 (9)	0.0149 (9)
C4	0.0490 (12)	0.0269 (10)	0.0258 (10)	0.0045 (9)	0.0149 (9)	0.0074 (8)
C5	0.0411 (12)	0.0405 (12)	0.0281 (10)	0.0028 (9)	0.0028 (9)	0.0148 (9)
C6	0.0354 (11)	0.0372 (11)	0.0302 (10)	0.0109 (9)	0.0074 (8)	0.0158 (9)
C7	0.0296 (10)	0.0264 (10)	0.0257 (9)	0.0065 (8)	0.0097 (7)	0.0131 (8)
C8	0.0306 (10)	0.0284 (10)	0.0292 (10)	0.0099 (8)	0.0104 (8)	0.0141 (8)
N9	0.0275 (8)	0.0271 (8)	0.0228 (7)	0.0075 (6)	0.0053 (6)	0.0112 (7)
C10	0.0369 (11)	0.0286 (10)	0.0265 (9)	0.0091 (8)	0.0104 (8)	0.0085 (8)
C11	0.0356 (10)	0.0274 (10)	0.0286 (10)	0.0052 (8)	0.0105 (8)	0.0078 (8)
N12	0.0306 (8)	0.0273 (8)	0.0256 (8)	0.0083 (7)	0.0081 (6)	0.0123 (7)
C13	0.0292 (10)	0.0307 (10)	0.0236 (9)	0.0038 (8)	0.0070 (7)	0.0126 (8)
C14	0.0259 (9)	0.0289 (10)	0.0257 (9)	0.0046 (7)	0.0071 (7)	0.0139 (8)
C15	0.0276 (10)	0.0385 (11)	0.0310 (10)	0.0070 (8)	0.0044 (8)	0.0180 (9)
C16	0.0325 (11)	0.0432 (12)	0.0438 (12)	0.0153 (9)	0.0114 (9)	0.0216 (10)
C17	0.0486 (13)	0.0348 (12)	0.0370 (11)	0.0171 (10)	0.0182 (10)	0.0121 (10)
C18	0.0365 (11)	0.0370 (11)	0.0257 (10)	0.0058 (9)	0.0051 (8)	0.0122 (9)
C19	0.0270 (9)	0.0318 (10)	0.0263 (9)	0.0081 (8)	0.0084 (7)	0.0162 (8)
N20	0.0297 (9)	0.0356 (9)	0.0307 (9)	0.0081 (7)	0.0022 (7)	0.0146 (8)

### Geometric parameters (Å, °)

**Table:**

N1—C2	1.464 (2)	C11—N12	1.490 (2)
N1—H1A	0.8900	C11—H11A	0.9700
N1—H1B	0.8900	C11—H11B	0.9700
N1—H1C	0.8900	N12—C13	1.499 (2)
C2—C3	1.382 (3)	N12—H12A	0.9000
C2—C7	1.383 (3)	N12—H12B	0.9000
C3—C4	1.376 (3)	C13—C14	1.502 (3)
C3—H3A	0.9300	C13—H13A	0.9700
C4—C5	1.377 (3)	C13—H13B	0.9700
C4—H4A	0.9300	C14—C15	1.392 (3)
C5—C6	1.378 (3)	C14—C19	1.393 (3)
C5—H5A	0.9300	C15—C16	1.374 (3)
C6—C7	1.389 (3)	C15—H15A	0.9300
C6—H6A	0.9300	C16—C17	1.380 (3)
C7—C8	1.501 (3)	C16—H16A	0.9300
C8—N9	1.503 (2)	C17—C18	1.381 (3)
C8—H8A	0.9700	C17—H17A	0.9300
C8—H8B	0.9700	C18—C19	1.378 (3)
N9—C10	1.484 (2)	C18—H18A	0.9300
N9—H9A	0.9000	C19—N20	1.462 (2)
N9—H9B	0.9000	N20—H20A	0.8900
C10—C11	1.510 (3)	N20—H20B	0.8900
C10—H10A	0.9700	N20—H20C	0.8900
C10—H10B	0.9700		
			
C2—N1—H1A	109.5	N12—C11—C10	110.36 (16)
C2—N1—H1B	109.5	N12—C11—H11A	109.6
H1A—N1—H1B	109.5	C10—C11—H11A	109.6
C2—N1—H1C	109.5	N12—C11—H11B	109.6
H1A—N1—H1C	109.5	C10—C11—H11B	109.6
H1B—N1—H1C	109.5	H11A—C11—H11B	108.1
C3—C2—C7	121.74 (18)	C11—N12—C13	111.41 (15)
C3—C2—N1	115.98 (17)	C11—N12—H12A	109.3
C7—C2—N1	122.25 (17)	C13—N12—H12A	109.3
C4—C3—C2	119.74 (19)	C11—N12—H12B	109.3
C4—C3—H3A	120.1	C13—N12—H12B	109.3
C2—C3—H3A	120.1	H12A—N12—H12B	108.0
C3—C4—C5	119.89 (19)	N12—C13—C14	112.95 (15)
C3—C4—H4A	120.1	N12—C13—H13A	109.0
C5—C4—H4A	120.1	C14—C13—H13A	109.0
C4—C5—C6	119.60 (19)	N12—C13—H13B	109.0
C4—C5—H5A	120.2	C14—C13—H13B	109.0
C6—C5—H5A	120.2	H13A—C13—H13B	107.8
C5—C6—C7	121.89 (19)	C15—C14—C19	117.02 (17)
C5—C6—H6A	119.1	C15—C14—C13	118.84 (17)
C7—C6—H6A	119.1	C19—C14—C13	124.07 (17)
C2—C7—C6	117.10 (17)	C16—C15—C14	121.56 (18)
C2—C7—C8	124.54 (17)	C16—C15—H15A	119.2
C6—C7—C8	118.31 (17)	C14—C15—H15A	119.2
C7—C8—N9	111.55 (16)	C15—C16—C17	120.13 (19)
C7—C8—H8A	109.3	C15—C16—H16A	119.9
N9—C8—H8A	109.3	C17—C16—H16A	119.9
C7—C8—H8B	109.3	C16—C17—C18	119.84 (19)
N9—C8—H8B	109.3	C16—C17—H17A	120.1
H8A—C8—H8B	108.0	C18—C17—H17A	120.1
C10—N9—C8	112.63 (15)	C19—C18—C17	119.46 (19)
C10—N9—H9A	109.1	C19—C18—H18A	120.3
C8—N9—H9A	109.1	C17—C18—H18A	120.3
C10—N9—H9B	109.1	C18—C19—C14	121.98 (18)
C8—N9—H9B	109.1	C18—C19—N20	117.28 (17)
H9A—N9—H9B	107.8	C14—C19—N20	120.72 (16)
N9—C10—C11	109.22 (16)	C19—N20—H20A	109.5
N9—C10—H10A	109.8	C19—N20—H20B	109.5
C11—C10—H10A	109.8	H20A—N20—H20B	109.5
N9—C10—H10B	109.8	C19—N20—H20C	109.5
C11—C10—H10B	109.8	H20A—N20—H20C	109.5
H10A—C10—H10B	108.3	H20B—N20—H20C	109.5
			
C7—C2—C3—C4	1.4 (3)	C10—C11—N12—C13	162.72 (16)
N1—C2—C3—C4	179.35 (17)	C11—N12—C13—C14	169.22 (16)
C2—C3—C4—C5	0.4 (3)	N12—C13—C14—C15	−84.1 (2)
C3—C4—C5—C6	−1.6 (3)	N12—C13—C14—C19	99.1 (2)
C4—C5—C6—C7	1.0 (3)	C19—C14—C15—C16	1.2 (3)
C3—C2—C7—C6	−1.9 (3)	C13—C14—C15—C16	−175.81 (19)
N1—C2—C7—C6	−179.74 (17)	C14—C15—C16—C17	−1.1 (3)
C3—C2—C7—C8	175.54 (18)	C15—C16—C17—C18	0.4 (3)
N1—C2—C7—C8	−2.3 (3)	C16—C17—C18—C19	0.2 (3)
C5—C6—C7—C2	0.7 (3)	C17—C18—C19—C14	−0.1 (3)
C5—C6—C7—C8	−176.90 (19)	C17—C18—C19—N20	178.41 (19)
C2—C7—C8—N9	105.5 (2)	C15—C14—C19—C18	−0.6 (3)
C6—C7—C8—N9	−77.2 (2)	C13—C14—C19—C18	176.23 (18)
C7—C8—N9—C10	−177.27 (15)	C15—C14—C19—N20	−179.02 (17)
C8—N9—C10—C11	172.08 (16)	C13—C14—C19—N20	−2.2 (3)
N9—C10—C11—N12	−175.50 (15)		

### Hydrogen-bond geometry (Å, °)

**Table:**

*D*—H···*A*	*D*—H	H···*A*	*D*···*A*	*D*—H···*A*
N1—H1A···Cl4	0.89	2.33	3.1067 (18)	146.
N1—H1B···Cl1^i^	0.89	2.30	3.1798 (18)	170.
N1—H1C···Cl2^ii^	0.89	2.26	3.1301 (18)	167.
N9—H9A···Cl1	0.90	2.21	3.1046 (17)	172.
N9—H9B···Cl2	0.90	2.22	3.0968 (17)	165.
N12—H12A···Cl3	0.90	2.18	3.0333 (18)	159.
N12—H12B···Cl2^iii^	0.90	2.35	3.1779 (17)	153.
N20—H20A···Cl1^iv^	0.89	2.29	3.1764 (19)	173.
N20—H20B···Cl3^iv^	0.89	2.56	3.2305 (18)	133.
N20—H20C···Cl3	0.89	2.23	3.1139 (18)	173.

Symmetry codes: (i) −*x*+1, −*y*+2, −*z*+1; (ii) *x*−1, *y*, *z*; (iii) −*x*+2, −*y*+1, −*z*+1; (iv) −*x*+1, −*y*+1, −*z*.
